# NKX2.2, PDX-1 and CDX-2 as potential biomarkers to differentiate well-differentiated neuroendocrine tumors

**DOI:** 10.1186/s40364-018-0129-8

**Published:** 2018-04-18

**Authors:** Michelle X. Yang, Ryan F. Coates, Abiy Ambaye, Valerie Cortright, Jeannette M. Mitchell, Alexa M. Buskey, Richard Zubarik, James G. Liu, Steven Ades, Maura M. Barry

**Affiliations:** 1 Department of Pathology and Laboratory Medicine, University of Vermont Medical Center, 111 Colchester Avenue, Burlington, VT 05401 USA; 2 Gastroenterology, University of Vermont Medical Center, Burlington, VT USA; 3 Applied Pathology Systems, Worcester, MA USA; 4 Medical Oncology, University of Vermont Medical Center, Burlington, VT USA

**Keywords:** Well-differentiated neuroendocrine tumor, Biomarker, NKX2.2, PDX-1, CDX-2, Gastrointestinal, Pancreas, Lung

## Abstract

**Background:**

Well-differentiated neuroendocrine tumors (NET) most frequently arise from the gastrointestinal tract (GI), pancreas, and lung. Patients often present as metastasis with an unknown primary, and the clinical management and outcome depend on multiple factors, including the accurate diagnosis with the tumor primary site. Determining the site of the NET with unknown primary remains challenging. Many biomarkers have been investigated in primary NETs and metastatic NETs, with heterogeneous sensitivity and specificity observed.

**Methods:**

We used high-throughput tissue microarray (TMA) and immunohistochemistry (IHC) with antibodies against a panel of transcriptional factors including NKX2.2, PDX-1, PTF1A, and CDX-2 on archived formalin-fixed paraffin-embedded NETs, and investigated the protein expression pattern of these transcription factors in 109 primary GI (*N* = 81), pancreatic (*N* = 17), and lung (*N* = 11) NETs.

**Results:**

Differential expression pattern of these markers was observed. In the GI and pancreatic NETs (*N* = 98), NKX2.2, PDX-1, and CDX-2 were immunoreactive in 82 (84%), 14 (14%), and 52 (52%) cases, respectively. PDX-1 was expressed mainly in the small intestinal and appendiceal NETs, occasionally in the pancreatic NETs, and not in the colorectal NETs. All three biomarkers including NKX2.2, PDX-1, and CDX-2 were completely negative in lung NETs. PTF1A was expressed in all normal and neuroendocrine tumor cells.

**Conclusions:**

Our findings suggest that NKX2.2 was a sensitive and specific biomarker for the GI and pancreatic neuroendocrine tumors. We proposed that a panel of immunostains including NKX2.2, PDX-1, and CDX-2 may show diagnostic utility for the most common NETs.

## Background

Gastrointestinal tract (GI), pancreas, and lung are among the most common sites to develop well-differentiated neuroendocrine tumors (NET). The incidence of GI NET is increasing, based on recent Surveillance, Epidemiology and End Results (SEER) data [1]. Patients with NET may initially present as liver metastasis in up to 19% of cases [2, 3], and the clinical management and outcome depend on multiple factors, including the primary tumor site [1, 4]. Although primary NET often shows a predominate architectural growth pattern at a particular site, tumor heterogeneity with mixed growth patterns do occur [5]. Using morphological features to diagnose the NET with an unknown primary can be challenging based on the hematoxylin and eosin staining alone, and a panel of immunohistochemistry (IHC) is often required to determine the site of primary tumor. General neuroendocrine markers including chromogranin and synaptophysin are very useful markers to determine the neuroendocrine differentiation. However, they lack specificity for the lineage or site of the tumor. Caudal type homeobox 2 (CDX-2) has been investigated previously by multiple groups and showed high sensitivity and specificity for small intestinal NET, but variable sensitivity for colorectal and pancreatic NETs. [6–9] PAX-8 and Islet-1 (ISL-1) have been investigated for their role to identify primary and metastatic pancreatic neuroendocrine tumors and variable sensitivity and specificity has been reported [10–13]. An extended panel of biomarkers including CDX-2, pancreatic and duodenal homeobox 1 (PDX-1), TTF-1, and NESP-55 has been reported in NETs from the pancreas, GI and lung, and these results also showed relatively low sensitivity for the tumors from the rectum and pancreas [14]. More recently, the expression pattern of CDX-2, PAX-6, ISL-1, ER, and PR in the GI and pancreatic NET was investigated and showed small differences between the primary and metastatic NETs [15, 16]. However, these markers showed relatively low sensitivity for pancreatic NETs. Investigation of more biomarkers to distinguish common NETs of different site remains necessary.

For this purpose, we focused on a panel of transcription factors (TFs) including CDX-2, NK2 homeobox 2 (NKX2.2), PDX-1, and pancreas specific transcription factor 1a (PTF1A) [17, 18], and simultaneously investigated their protein expression pattern in the NETs of the most common sites, including GI, pancreas and lung. PDX-1 is a transcription factor essential for both pancreatic ductal and islet cell development during early embryogenesis [18]. After development, PDX-1 is predominantly expressed in the islet cells of pancreas, but not in the ductal epithelium or acinar cells [19]. Interestingly, NKX2.2 is critical for the differentiation of beta cells in the islet cells of the pancreas [20–26], and important for the differentiation of enteroendocrine cells of the intestine [27, 28], as well as oligodendrocytes of the central nervous system [29, 30]. During early pancreatic development, NKX2.2 protein expression is initiated with PDX-1 and PTF1A in the dorsal and ventral pancreatic buds [31]. In the intestine, NKX2.2 is expressed in the endocrine cells located in the villi and deep crypts, and a rare subset of NKX2.2-positive cells is coexpressed with intestinal stem cell markers [32, 33]. We compared the results with the well-known transcriptional factor of intestinal differentiation, CDX-2.

## Methods

### Study population

A total of 109 primary NETs were retrospectively retrieved from archived blocks at our institution from January 2010 to December 2015, including 92 resections and 17 biopsies. The tumor grade for all cases was extracted from original pathological report for all NETs, based on current World Health Organization (WHO) criteria for each tumor site on the H&E stain. Particularly, 11 lung NETs included 7 typical carcinoids and 4 atypical carcinoids based on 2015 WHO criteria for lung NETs. All the slides were reviewed by two pathologists and representative blocks were selected for this study. This study was approved by our Institutional Review Board (IRB #CHRMS 17–0009).

### Tissue microarray (TMA) and immunohistochemistry (IHC)

Using duplicated 2-mm core punch (Beecher Instruments Inc., Sun Prairie, WI), TMA was constructed from formalin-fixed and paraffin-embedded tumor or normal blocks for resection cases, and 2–3 levels of whole tissue section were mounted on the slides for all biopsies. PDX-1 (clone 2A12, 1:100 dilution, Abcam, Cambridge, MA), PTF1A (clone 1A2, 1:200 dilution, Abcam), and NKX2.2 (clone NX2/294, 1:100 dilution, Abcam) immunohistochemistry was initially validated in normal pancreatic tissues. CDX-2 (clone EP25, Leica Biosystems, Buffalo Grove, IL) has been validated according to the College of American Pathologists (CAP) guidelines in the histology lab for routine clinical use. Antigen retrieval was obtained as follows: CDX-2 in H2 buffer (Leica Biosystems) for 10 min, NKX2.2 and PTF1A in H1 buffer (Leica Biosystems) for 30 min, and PDX-1 in H1 for 10 min. All IHCs were performed on BOND-III automated IHC stainer (Leica Biosystems).

## Results

### Demographics and tumor site

Among the 109 patients, 51 were males and 58 were females with a median age of 57 years (ranging from 12 to 90 years). In this study population, the tubular GI tract was the most common site for NET, followed by the pancreas and lung (Table 1). For 11 lung NETS, 7 cases were typical carcinoid and 4 cases were atypical carcinoid tumors.

**Table 1:** List of well-differentiated neuroendocrine tumors used in this study

Site	Grade 1	Grade 2	Total No.
Rectum	15	1	16
Colon	4	1	5
Appendix	20	2	22
Ileum	11	1	12
Jejunum	4	6	10
Duodenum	3	1	4
Stomach	11	1	12
Pancreas	12	5	17
Total Number	80	18	98

### Expression of NKX2.2, PDX-1 and PTF1A in normal GI and pancreaticobiliary endocrine cells

**Fig. 1:**
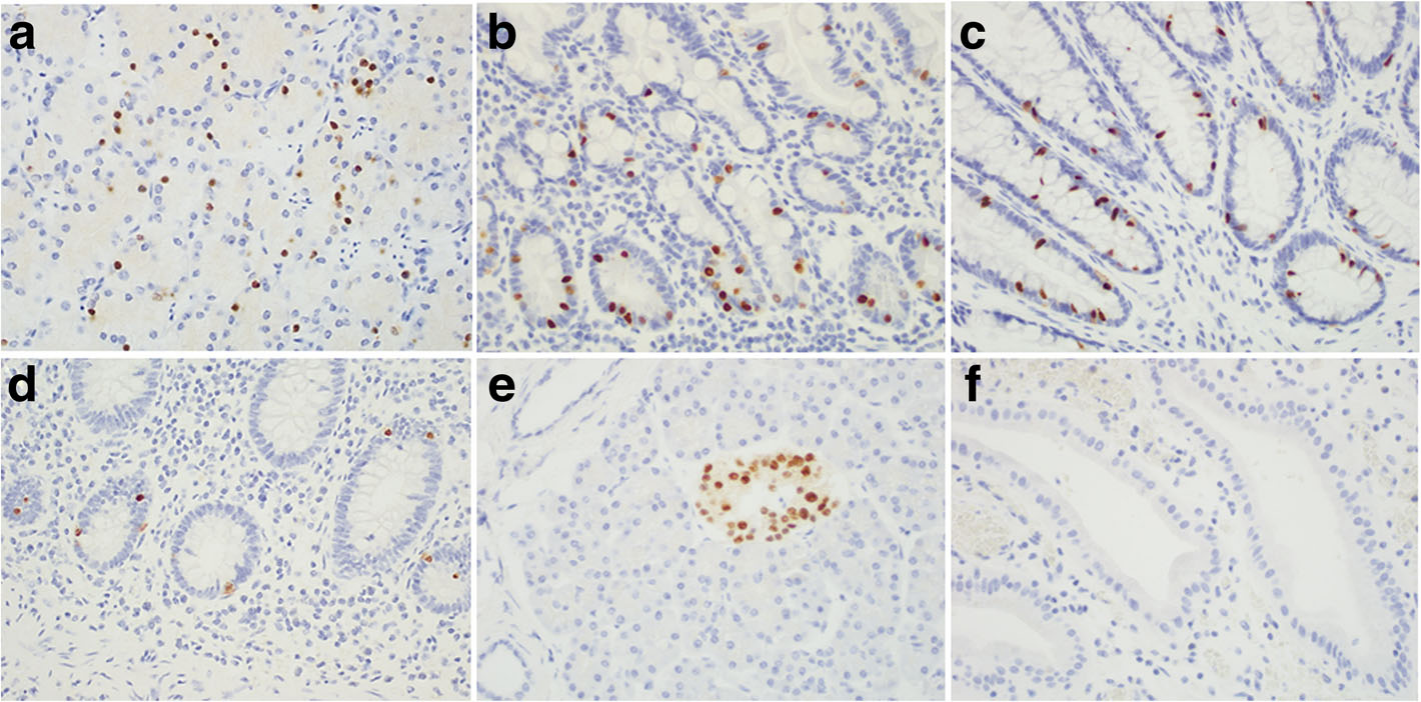
NKX2.2 immunoreactivity in endocrine cells of normal stomach (**a**), duodenum (**b**), colon (**c**), appendix (**d**), and pancreas (**e**). No NKX2.2 positive cells identified in the gallbladder (**f**). Original magnification, 400×

In normal GI tract, scattered NKX2.2 immunoreactive nuclei were observed in the endocrine cells predominantly located in the deep crypts of the entire tubular GI tract, including stomach, duodenum, jejunum, ileum, appendix, colon, and rectum (Fig. 1a, d). No nuclear staining for PDX-1 was seen in the tubular GI tract. PTF1A was immunoreactive in all normal GI tract mucosa.

In normal pancreas, both NKX2.2 (Fig. 1e) and PDX-1 (data not shown) were expressed in the nucleus of islet cells, and were completely negative in normal ductal epithelium and acinar cells. In normal gallbladder, no NKX2.2 or PDX-1 immunoreactive cells were detected (Fig. 1f). PTF1A expression was seen in all types of parenchymal cells in the pancreas (data not shown).

### NKX2.2, PDX-1 and CDX-2 expression in NETs of the GI tract

Although IHC interpretation was defined as positive if > 5% of tumor cells had nuclear staining for NKX2.2, CDX-2, PDX-1 and PTF1A, the majority of positive cases demonstrated diffuse nuclear immunoreactivity in both TMA and biopsy cases, and focal or patchy staining in rare cases were seen only in the gastric and pancreatic NETs. We performed IHC on whole sections of 8 of 92 resection cases (8.7%) of the NETs from the GI tract and pancreas, and all positive and negative results were consistent with those on TMA with either diffuse positivity or completely negativity.

**Fig. 2:**
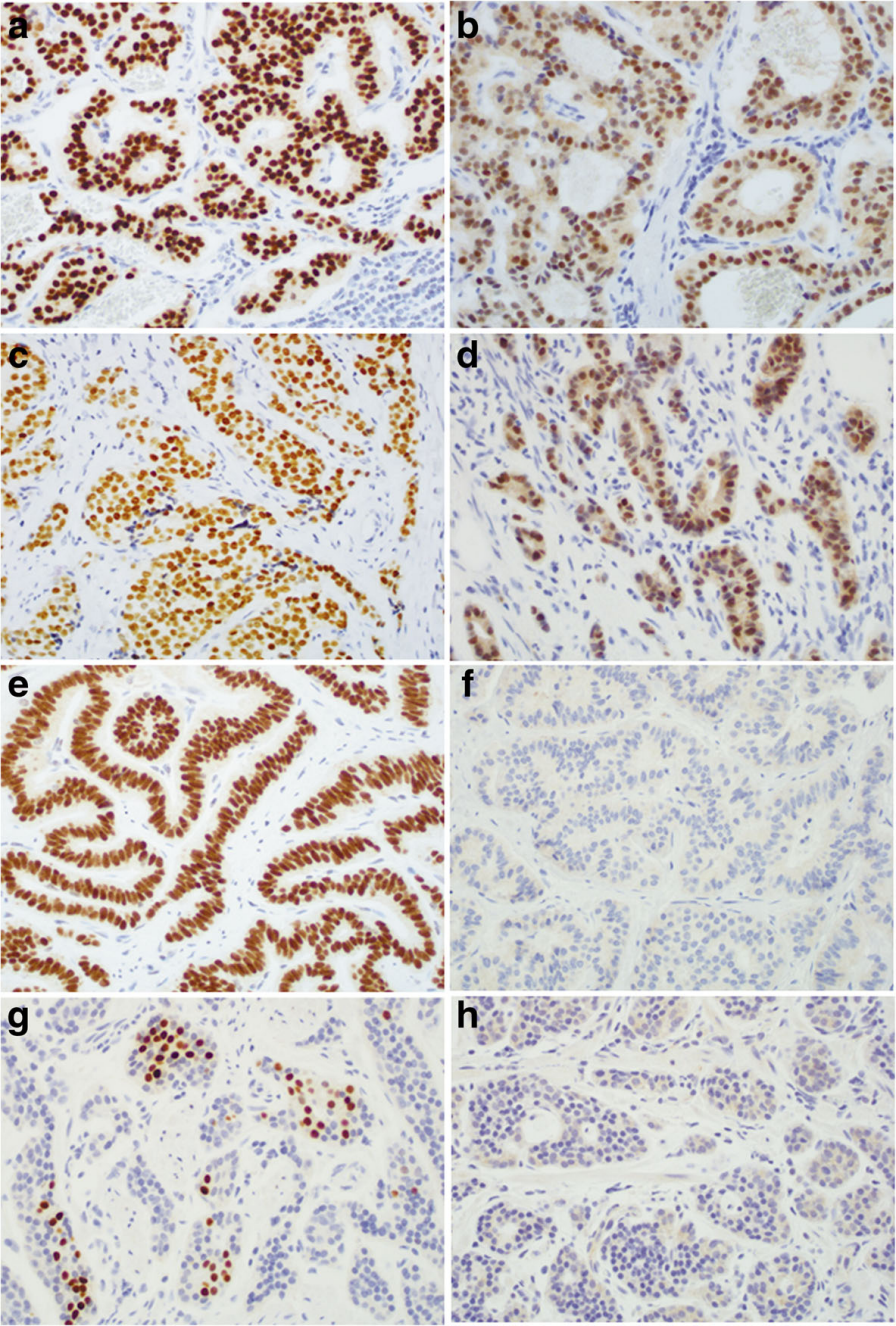
Representative immunostain of NKX2.2 and PDX-1 in well-differentiated neuroendocrine tumor of the duodenum (**a**, **b**), appendix (**c**, **d**), colorectum (**e**, **f**), and stomach (**g**, **h**), respectively. Original magnification, 400×

**Fig. 3:**
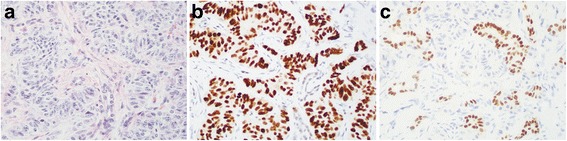
Diffuse immunoreactivity of NKX2.2 compared to patchy immunoreactivity of CDX-2 in representative pancreatic well-differentiated neuroendocrine tumors. **a** H&E staining. **b** NKX2.2. **c** CDX-2. Original magnification, 400×

In 98 GI and pancreatic NETs, the sensitivity and 95% confidence interval (95% CI) for NKX2.2, PDX-1, and CDX-2 were 84% (95% CI, 75% - 90%), 14% (95% CI, 8% - 23%), and 53% (95% CI, 43% - 63%), respectively. There was no significant difference in all 3 biomarkers between G1 and G2 tumors. Along the GI tract, NKX2.2 immunoreactivity was strong and diffuse in the tumor nuclei of the majority of NETs along the GI tract (Table 2; Fig. 2). Although NKX2.2-positive endocrine cells were seen in normal gastric pits as shown in Fig. 1a, the majority of gastric NETs showed negative and only rare cases with patchy immunoreactivity for NKX2.2 (Fig. 2g). For PDX-1, immunoreactivity was seen in 75% duodenum, 32% appendix, 20% jejunum, 8% ileum, and 6% pancreas NETs (Table 2; Fig. 2). None of the gastric, colonic, and rectal NETs was immunoreactive to PDX-1 (Table 2 and Fig. 2). NKX2.2 IHCs were repeated on all gastric NETs and results remained the same. For 17 pancreatic NETs, NKX2.2 was diffusely immunoreactive in 71% cases (Table 2 and Fig. 3a, b). However, PDX-1 was immunoreactive in only 1 of 17 pancreatic NETs.

**Table 2:** Immunoreactivity of NKX2.2, PDX-1 and CDX-2 in 109 well-differentiated neuroendocrine tumors

Site	Total = 109	NKX2.2 +	PDX-1 +	CDX-2 +
Rectum	16	16 (100%)	0	0
Colon	5	5 (100%)	0	3 (60%)
Appendix	22	22 (100%)	7 (32%)	22 (100%)
Ileum	12	11 (92%)	1 (8%)	12 (100%)
Jejunum	10	10 (100%)	2 (20%)	10 (100%)
Duodenum	4	4 (100%)	3 (75%)	3 (75%)
Stomach	12	2 (17%)	0	0
Pancreas	17	12 (71%)	1 (6%)	2 (11%)
Lung	11	0	0	0
Total GI-pancreas	98	82 (84%)	14 (14%)	52 (53%)

Abbreviation: GI, gastrointestinal

As expected, CDX-2 was diffusely positive in all 22 appendiceal, 96% small intestinal, and 60% of colonic NETs. Consistent with the literature, no immunoreactivity for CDX-2 was seen in all 16 rectal NETs, and only 2 of 17 pancreatic NETs showed patchy immunoreactivity for CDX-2 (Table 2 and Fig. 3c). In other words, both CDX-2 and NKX2.2 showed similarly high immunoreactivity rate in the appendiceal, ileal and jejunum NETs. For pancreatic, duodenal, colonic and rectal NETs, NKX2.2 showed much higher immunoreactivity than CDX-2. All three biomarkers including NKX2.2, PDX-1, and CDX-2 were completely negative in all 11 lung NETs (Table 2).

### Non-specific PTF1A expression pattern in all NETs

Unlike the NKX2.2, PDX-1, and CDX-2 that had restricted expression pattern in NETs of different site, PTF1A was expressed in all normal and neuroendocrine tumor cells (data not shown). Therefore, PTF1A appeared to be a non-specific biomarker for all NETs, although a different source of antibody may be tested before a final conclusion can be made.

## Discussion

In this study, four transcriptional factors including CDX-2, NKX2.2, PDX-1, and PTF1A were simultaneously investigated for their protein expression in the most common NETs from the GI, pancreatic, and lung by IHC. We found that these transcription factors were differentially expressed in NETs at these anatomic sites. NKX2.2 was a highly sensitive biomarker for neuroendocrine differentiation of pancreatic and intestinal neuroendocrine origin. PDX-1 was expressed in NETs of small intestine and appendix with variable but higher immunoreactivity at these sites, but not in the gastric, colonic or rectal NETs. NKX2.2 was positive in over 2/3 pancreatic NETs. Although PDX-1 immunopositivity was seen in less than 1/3 pancreatic NETs in previous reports, it was positive in 6% of pancreatic NETs in this study probably due to different source of antibodies, since it was positive in all internal controls. Although PTF1A was characterized as one of the pancreatic lineage-specific transcription factors (TFs), its expression was non-specific and was not a useful biomarker for NETs. Consistent with other’s results, CDX-2 can be a useful biomarker for NETs arising from the small intestine and appendix. However, it showed only 50% immunoreactivity in colonic NETs and no immunoreactivity in all rectal NETs. CDX-2 was patchy positive in 11% of pancreatic NETs similarly seen in a previous study [14]. Overall, NKX2.2 showed much higher sensitivity for both rectal and pancreatic NETs, compared to PDX-1 and CDX-2 for the tumors at these sites. Most importantly, these biomarkers were completely negative in all lung NETs, pancreatic ductal adenocarcinomas, or other carcinomas (data not shown).

**Fig. 4:**
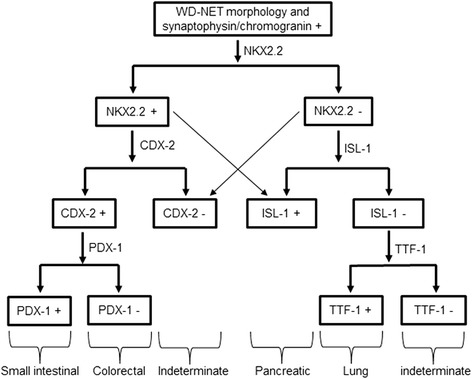
Proposed diagnostic algorithm for WD-NETs

Previous studies showed that NKX2.2 was negative in 13 bronchopulmonary carcinoid tumors, whereas 24 of 26 GI carcinoids (including 1 stomach, 1 duodenum, 2 ampullary, 14/16 pancreas, 5 ileum, and 1 colon), were immunoreactive for NKX2.2 [34]. In this study, we argue that a panel of PDX-1, CDX-2, and NKX2.2 would significantly increase the sensitivity of NETs from the GI tract and pancreas. Although TTF-1 was not tested in this cohort, it has been studied to determine NETs of pulmonary origin by several groups [8, 35–38]. Considering the fact that the majority of neuroendocrine tumors arise from the tubular GI tract, pancreas, and lung, we proposed a diagnostic algorithm using a panel of IHC stains including NKX2.2, PDX-1, CDX-2, and TTF-1 would aid the diagnosis of the primary tumor site in the setting of metastasis (Fig. 4). Although ISL-1, PAX-6, and PAX-8, were not investigated in the primary NETs in our study, they can be added into the panel when the results are indeterminate for the pancreatic tumor or to increase the confidence of prediction [12], although the specificity of PAX-8 and ISL-1 was variable in different studies [39, 40]. Interestingly, a recent study showed high sensitivity of NKX2.2 in 85 liver metastatic NETs of the digestive system [41].

## Conclusions

Using IHC, we found differential immunoreactivity of NKX2.2, CDX-2, and PDX-1 to the NETs from a different anatomic site. NKX2.2 showed higher sensitivity than CDX-2 and PDX-1 in both GI and pancreatic NETs. PDX-1 was expressed mainly in the small intestinal and appendiceal NETs, occasionally in the pancreatic NETs, and not in the colorectal NETs. Importantly, NKX2.2, CDX-2, and PDX-1 were not expressed in 11 lung NETs, and we understand the limitation of small sample size for a final conclusion on the specificity of these markers. These findings suggest that a panel of biomarkers including NKX2.2, PDX-1, and CDX-2 may provide additional insights in clinical diagnosis for the most common NETs especially in the setting of metastasis with an unknown origin.

## Abbreviations

CDX-2: Caudal type homeobox 2
ER: Estrogen receptor
GI: Gastrointestinal tract3
IHC: Immunohistochemistry
ISL-1: Islet 1
NET: Well-differentiated neuroendocrine tumors
NKX2.2: NK2 homeobox 2
PAX-6: Paired box gene 6
PAX-8: Paired box gene 8
PDX-1: Pancreatic and duodenal homeobox 1
PR: Progesterone receptor
PTF-1A: Pancreas specific transcription factor 1a
SEER: Surveillance, Epidemiology and End Results
TF: Transcription factor
TMA: Tissue microarray
TTF-1: Transcription termination factor 1
WHO: World Health Organization
